# Supplementary material for: Comparative analysis of 10X Chromium vs. BD Rhapsody whole transcriptome single-cell sequencing technologies in complex human tissues
Source: Heliyon. 2024 Mar 19;10(7):e28358. doi: 10.1016/j.heliyon.2024.e28358 (PMC11059509; doi:10.1016/j.heliyon.2024.e28358)
Supplement: Multimedia component 1 [file mmc1.docx]

Supplemental information

Comparative analysis of 10X Chromium vs. BD Rhapsody whole transcriptome

single-cell sequencing technologies in complex human tissues

Stefan Salcher, Isabel Heidegger, Gerold Untergasser, Georgios Fotakis, Alexandra Scheiber, Agnieszka Martowicz, Asma Noureen, Anne Krogsdam, Christoph Schatz, Georg Schäfer, Zlatko Trajanoski, Dominik Wolf, Sieghart Sopper and Andreas Pircher

**Figure S1: related to Figure 1: scRNAseq workflow.**

(A) Workflow of 10X Chromium and BD Rhapsody scRNAseq protocols form cell preparation to sequencing library preparation. The first “stopping point” is indicated.

**Figure S2: related to Figure 2: QC metrics in datasets generated with 10X Chromium and BD Rhapsody.**

(A) nFeature, nCount, and %MT quality metrics in unfiltered individual samples processed with 10X Chromium and BD Rhapsody.

(B) nFeature, nCount, and %MT quality metrics after filtering for high quality cells (nCounts > 2000, nFeatures > 200 and < 8000, %MT < 30%) in individual samples processed with 10X Chromium and BD Rhapsody.

**Figure S3: related to Figure 2: Transcripts associated with RNA decay.**

(A) Expression of transcripts associated with RNA decay in 10X Chromium and BD Rhapsody data generated from benign prostate and PCa samples.

**Figure S4: related to Figure 3: Prostate cancer TME revealed by 10X Chromium and BD Rhapsody.**

(A) Uniform manifold approximation and projection (UMAP) plot of 26,266 high-quality cells, color-coded by individual samples.

(B) UMAP plot colored by cells derived from benign and PCa (tumor) tissues.

(D-F) UMAP plot colored by cell type (D) and by individual samples (E) in data generated with BD Rhapsody.

**Figure S5: related to Figure 4: Molecule capture efficiency and sequencing library complexity.**

(A) Gene expression levels of indicated house-keeping genes in raw sequencing data generated with 10X Chromium and BD Rhapsody

(B and C) Gene expression levels of indicated house-keeping genes in raw sequencing data (B) and normalized data (C) in individual samples.

**Figure S6: related to Figure 4: Expression of lncRNAs in NSCLC single-cell atlas.**

(A) Gene expression levels of the lncRNAs *MALAT1* and *NEAT1* in individual cell types of the prostate TME depicted with 10X Chromium and BD Rhapsody.

(B) Gene expression levels of the lncRNAs *MALAT1* and *NEAT1* in cell types depicted in the NSCLC single-cell atlas (Salcher et al., *Cancer Cell* 2022). Datasets generated with 10X Chromium and BD Rhapsody platforms have been analyzed.

(C) Gene expression levels of the lncRNAs *MALAT1* and *NEAT* in the NSCLC single-cell atlas (Salcher et al., *Cancer Cell* 2022) indatasets generated with 10X Chromium and BD Rhapsody.

**Figure S7: related to Figure 5: Platform-specific gene quantification and cell-type marker identification.**

(A) Gene expression levels of *IFI27*, *VWF*, and *CD34* in raw sequencing data detected in endothelial cells by 10X Chromium and BD Rhapsody.

(B) Gene expression levels of the lncRNAs *MALAT1* and *NEAT.* Each dot refers to a sample (benign or tumor tissue) with at least 40 endothelial cells in both 10X Chromium and BD Rhapsody groups. Paired t-test, ^*^p ≤ 0.05.

**Figure S8: related to Figure 6: Platform-dependent cellular composition in scRNA-seq data.**

(A) The proportion of NK cells, B cells, plasma cells, macrophages, mast cells, as well as endothelial cells depicted by 10X Chromium vs. BD Rhapsody in individual samples. Paired t-test.

(B) Proportion of mitochondrial mitochondrial transcripts (%MT) in individual cell types depicted with 10X Chromium and BD Rhapsody.
